# Neuroimaging Anomalies in Community-Dwelling Asymptomatic Adults With Very Early-Stage White Matter Hyperintensity

**DOI:** 10.3389/fnagi.2021.715434

**Published:** 2021-08-16

**Authors:** Shuai Guan, Xiangyu Kong, Shifei Duan, Qingguo Ren, Zhaodi Huang, Ye Li, Wei Wang, Gaolang Gong, Xiangshui Meng, Xiangxing Ma

**Affiliations:** ^1^Department of Radiology, Qilu Hospital (Qingdao), Cheeloo College of Medicine, Shandong University, Qingdao, China; ^2^State Key Laboratory of Cognitive Neuroscience and Learning and IDG/McGovern Institute for Brain Research, Beijing Normal University, Beijing, China; ^3^Department of Radiology, Qingdao Central Hospital, Qingdao University, Qingdao, China

**Keywords:** white matter hyperintensity, cerebral small vessel disease, cortical thickness, diffusion tensor imaging, functional magnetic resonance imaging

## Abstract

White matter hyperintensity (WMH) is common in healthy adults in their 60s and can be seen as early as in their 30s and 40s. Alterations in the brain structural and functional profiles in adults with WMH have been repeatedly studied but with a focus on late-stage WMH. To date, structural and functional MRI profiles during the very early stage of WMH remain largely unexplored. To address this, we investigated multimodal MRI (structural, diffusion, and resting-state functional MRI) profiles of community-dwelling asymptomatic adults with very early-stage WMH relative to age-, sex-, and education-matched non-WMH controls. The comparative results showed significant age-related and age-independent changes in structural MRI-based morphometric measures and resting-state fMRI-based measures in a set of specific gray matter (GM) regions but no global white matter changes. The observed structural and functional anomalies in specific GM regions in community-dwelling asymptomatic adults with very early-stage WMH provide novel data regarding very early-stage WMH and enhance understanding of the pathogenesis of WMH.

## Introduction

White matter hyperintensity (WMH) (also widely referred to as leukoaraiosis) is a cerebral small vessel disease that shows hyperintensity on T2-weighted and fluid attenuated inversion recovery (FLAIR) MR images but isointensity or hypointensity on T1-weighted images (Wardlaw et al., 2013). WMH patients can be clinically asymptomatic or symptomatic, such as cognitive decline and physical disability. The underlying pathogenesis of WMH remains unclear and is putatively multifactorial (Wardlaw et al., 2013, 2019).

There have been numerous MRI studies on symptomatic patients with late-stage WMH, which demonstrated various structural and functional abnormalities across the brain in patients compared with healthy controls (O’Sullivan et al., 2001; O’Sullivan, 2004; Nitkunan et al., 2008; Seo et al., 2010; Li et al., 2012, 2015; Yi et al., 2012; Lawrence et al., 2013, 2014; Kim et al., 2014; Mascalchi et al., 2014; Lambert et al., 2015; Benjamin et al., 2016; Chen et al., 2016, 2019; Wang et al., 2019a; Wang et al., 2019b; Zhu et al., 2020). For example, gray matter (GM) atrophy and functional changes in the default mode network have been reported in subcortical vascular mild cognitive impairment (MCI) patients with WMH (Yi et al., 2012). Altered diffusion MRI-derived parameters [fractional anisotropy (FA) and mean diffusivity (MD)] have been found in cognitively impaired patients with WMH (Lawrence et al., 2013). On the other hand, a few studies have looked at asymptomatic patients with WMH, but the patients’ stage of WMH was not controlled for Wen et al. (2006); Reijmer et al. (2015); Keřkovský et al. (2019). Additionally, there were limitations in these studies regarding the selection of clinically asymptomatic patients, e.g., using simple questionnaires, possibly including patients with MCI (Keřkovský et al., 2019), or not excluding patients with a history of chronic stroke (Wen et al., 2006).

To date, it remains unknown whether and how asymptomatic patients with very early-stage WMH (e.g., with a Fazekas grade 1) (Fazekas et al., 1987) differ in neuroimaging-based brain structural and functional profiles from non-WMH controls, which will provide insight into the pathogenesis underlying very early-stage WMH. To address this, the present study recruited a group of community-dwelling adults with a Fazekas grade 1 for WMH who were strictly asymptomatic in terms of neurological scales. Multimodal MRI (structural, diffusion, and resting-state functional MRI) measures were compared between this group and an age-, sex-, and education-matched non-WMH control group.

## Materials and Methods

### Participants

All subjects were recruited from community. All participants signed an informed consent form, and the study was approved by our institutional ethical committee. Neuropsychological assessments of all participants were evaluated using the Mini-Mental State Examination (MMSE) and activities of daily living scale (ADL). Participants were entered into our analysis if they met the following criteria: (1) 40–65 years old, (2) MMSE score ≥27, ADL score = 14, (3) mild WMH located in periventricular or deep white matter (WM) on T2-weighted and FLAIR sequences, (4) no history of drug use that could have affected cognitive function, (5) right-handed, and (6) capable of completing the MRI examination with a qualifying high-resolution MRI image. Participants were excluded if they met the following criteria: (1) a history of frequent dizziness and headache; (2) a history of severe systemic diseases, such as cardiovascular, liver, or kidney diseases; (3) a history of cerebral infarction, cerebral hemorrhage, brain tumor brain surgery or trauma of the brain; (4) acute or chronic cerebral infarction, lacune, microbleed, tumor, infectious disease, or metabolic disease detected by MRI; (5) a history of alcohol or drug dependence during the last 6 months; and (6) a history of intracranial metal implantation.

### Data Acquisition

All data were acquired on a 3T MRI scanner (Ingenia, Philips Medical Systems, Netherlands). The matched head coil was used with foam padding and earplugs to reduce head motion and scanner noise. Conventional T1-weighted (repetition time (TR) = 2000 ms, echo time (TE) = 20 ms, flip angle (FA) = 90°, field of view (FOV) = 230 mm × 230 mm, data matrix = 288 × 180, slice thickness (ST) = 6 mm, gap = 1 mm, 18 slices, number of signals averaged (NSA) = 1, orientation: transverse), T2-weighted (TR = 2369 ms, TE = 107 ms, FA = 90°, FOV = 230 mm × 230 mm, data matrix = 352 × 352, ST = 6 mm, gap = 1 mm, 18 slices, NSA = 1, orientation: transverse), FLAIR (TR = 7000 ms, TE = 125 ms, FA = 90°, FOV = 230 mm × 230 mm, data matrix = 288 × 163, ST = 6 mm, gap = 1 mm, 18 slices, NSA = 1, orientation: transverse), DWI (TR = 2235 ms, TE = 76 ms, FA = 90°, FOV = 230 mm × 230 mm, data matrix = 176 × 134, ST = 6 mm, gap = 1 mm, 18 slices, NSA = 1, orientation: transverse), three-dimensional T1-weighted (TR = 6.7 ms, TE = 3.0 ms, FA = 8°, FOV = 240 mm × 240 mm, data matrix = 240 × 240, ST = 1 mm, gap = 0 mm, 170 slices, NSA = 1, orientation: sagittal), DTI (TR = 4900 ms, TE = 95 ms, FA = 90°, FOV = 224 mm × 224 mm, data matrix = 112 × 110, ST = 2 mm, gap = 0 mm, 70 slices, NSA = 2, 33 non-collinear directions, *b* = 1000 s/mm^2^, orientation: transverse), and resting-state functional (TR = 2000 ms, TE = 30 ms, FA = 90°, FOV = 230 mm × 230 mm, data matrix = 68 × 66, ST = 4 mm, gap = 0.5 mm, 32 slices, 240 time points, NSA = 1, orientation: transverse) images were acquired using the same 3T Philips scanner.

**FIGURE 1:**
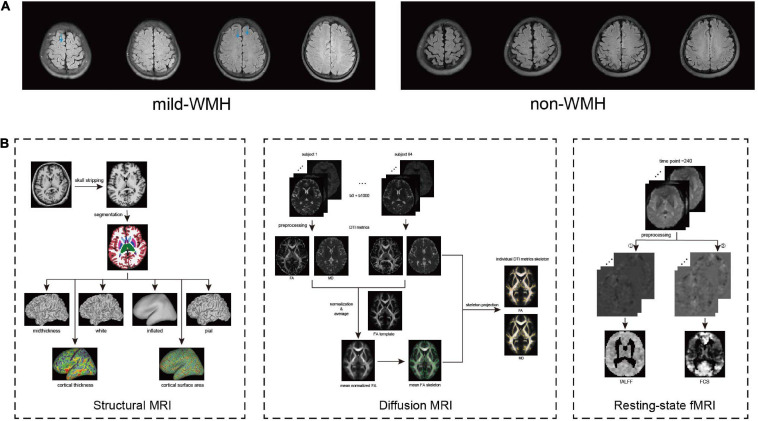
**(A)** The example image from the mild-WMH and non-WMH control groups. **(B)** The workflow of process of 3D T1, DTI, and resting-state functional images.

Forty-seven subjects were included in the mild-WMH group (Figure 1A). Thirty-seven age-, sex-, and education-matched normal subjects were recruited as the non-WMH control group. All 84 subjects had a high-quality three-dimensional T1-weighted image and were entered into the T1-related analysis. Eight mild-WMH and four non-WMH subjects were excluded from the DTI-related analysis due to poor DTI image quality. Ten mild-WMH subjects and eight non-WMH subjects were excluded from the resting-state fMRI analysis due to poor fMRI image quality. The demographic characteristics of the subjects are detailed in Table 1.

**TABLE 1:** The demographic characteristics of the subjects.

	**3D T1**			**DTI**			**rs-fMRI**		
	**Mild-WMH (*n* = 47)**	**Non-WMH (*n* = 37)**	***P*-value**	**Mild-WMH (*n* = 39)**	**Non-WMH (*n* = 33)**	***P*-value**	**Mild-WMH (*n* = 37)**	**Non-WMH (*n* = 29)**	***P*-value**
Age, years	49.8 (5.8)	48.1 (6.6)	0.205*	50.2 (5.6)	47.9 (6.2)	0.104*	49.8 (5.7)	48.0 (6.5)	0.248*
Sex: female, *n* (%)	31 (66.0%)	25 (67.6%)	0.876**	26 (66.7%)	21 (63.6%)	0.788**	26 (70.3%)	19 (65.5%)	0.681**
Education, years	13.3 (3.3)	13.6 (4.1)	0.710*	13.3 (2.9)	13.6 (4.3)	0.678*	13.1 (3.0)	13.6 (4.3)	0.567*
MMSE	29.0 (0.9)	29.2 (0.9)	0.419*	29.1 (0.9)	29.2 (1.0)	0.659*	29.1 (0.9)	29.3 (0.9)	0.322*
ADL	14 (0)	14 (0)	1.000*	14 (0)	14 (0)	1.000*	14 (0)	14 (0)	1.000*
Hypertension, *n* (%)	11 (23.4%)	7 (18.9%)	0.619**	9 (23.1%)	6 (18.2%)	0.610**	10 (27.0%)	5 (17.2%)	0.346**
DM, *n* (%)	3 (6.4%)	4 (10.8%)	0.740***	3 (7.7%)	4 (12.1%)	0.816***	3 (8.1%)	3 (8.1%)	0.906***
Hyperlipidemia, *n* (%)	8 (17.0%)	5 (13.5%)	0.659**	7 (17.9%)	5 (17.9%)	0.751**	8 (21.6%)	4 (13.8%%)	0.413**
Current smoker, *n* (%)	3 (6.4%)	4 (10.8%)	0.740***	2 (5.1%)	4 (12.1%)	0.521***	1 (2.7%)	4 (13.8%)	0.222***
Current drinker, *n* (%)	14 (29.8%)	15 (40.5%)	0.303**	11 (28.2%)	14 (42.4%)	0.207**	10 (27.0%)	11 (27.0%)	0.345**

*Two-sample *t*-tests, **χ^2^ test, ***adjusted χ^2^ test.

To estimate the location and size of WMH for each subject, we manually checked the WMH on the MRI image by an experienced radiologist. Specifically, the location was categorized into periventricular, frontal lobe, parietal lobe, temporal lobe, occipital lobe, insular lobe, and subcortical nuclei. Given the very small volume of each WMH, we simply used the number of WMH as the WMH size. The vast majority of WMHs are located in the frontal lobe across all subjects, and there was no significant correlation between the WMH size and age across the patients.

### MRI Data Processing

Processing workflow of the three modalities (structural, diffusion, and resting-state functional MRI) are detailed in Figure 1B. Structural images were processed using FreeSurfer (Fischl, 2012). All participants’ T1-weighted images were processed with an automatic FreeSurfer “recon-all” pipeline. Briefly, the implemented processing stream involved removal of non-brain tissue; transformation to the Talairach reference space; segmentation into GM and WM; correction of topological defects; intensity normalization; and sub-voxel representation of the GM/WM boundary and pial surfaces. In short, the FreeSurfer pipeline generates models of the individual cortical surface with sub-voxel/sub-millimeter precision, yielding measures of cortical thickness and surface area at each vertex of the surface (Fischl and Dale, 2000). Cortical thickness was defined by the shortest distance between the GM/WM border and pial surfaces, while surface area was calculated as the mean area of the triangular region at the vertex. Then, all surface images were normalized to fsaverage space with the function “mris_preproc” in FreeSurfer and subsequently smoothed using a Gaussian kernel with a 20-mm FWHM (Seo et al., 2012).

DTI data preprocessing and analyses were performed using PANDA (Pipeline for Analyzing Brain Diffusion Images toolkit) software (Cui et al., 2013).^1^ Correction for head movement and eddy distortion and removal of non-brain structures were performed. The tensor image parameters FA and MD were generated for each subject. The FA image of each subject was registered into the standard space through non-linear registration. The registered FA images were averaged, and the average FA images were used to extract the WM FA skeleton. A group of WM FA skeletons were projected onto two tensor index (FA and MD) images of each subject in standard space to obtain WM skeleton images with different parameters. The Randomize tool of the Oxford University Functional Magnetic Resonance Imaging Research Centre software library (FSL^2^) was used for data analysis of whole brain tract-based spatial statistics (TBSS) at the voxel level.

Preprocessing of the resting-state functional images was performed using the Data Processing & Analysis of Brain Imaging (DPABI^3^) toolkits. Most of the toolkit functions in DPABI are based on Statistical Parametric Mapping (SPM12).^4^ The first 10 time points were removed for signal equilibrium and participant adaptation to the scanning noise. Then, slice timing and head motion correction were performed. Then, a group-wise Diffeomorphic Anatomical Registration Through Exponentiated Lie Algebra (DARTEL) template (Ashburner, 2007) was created for spatial normalization to the standard Montreal Neurological Institute 152 space and resampled to 3 mm × 3 mm × 3 mm. The normalized images were smoothed with a three-dimensional isotropic Gaussian kernel with a 4-mm FWHM. Linear detrending was then applied to reduce low-frequency drifts. Covariates were regressed out from the time series of every voxel. The covariates included a total of 26 variables, including the WM signal, the cerebrospinal fluid (CSF) signal, and the Friston 24-parameter model. The Friston 24-parameter model of head motion includes the six standard head motion parameters, the derivative of the standard motion parameters to account for a one-frame delay in the effect of motion on the blood oxygenation level-dependent (BOLD) signal and the 12 corresponding squared items. Additionally, a band-pass temporal filter (0.01–0.1 Hz) was applied to reduce high-frequency physiological noise. Finally, based on the record of head motions within each fMRI run, the volume-to-volume framewise displacement (FD) was calculated for scrubbing. Any volume with FD >0.5 mm (i.e., the FD-flagged volumes) and volumes 1 back (time point before FD-flagged volumes) and 2 forward (time point after FD-flagged volumes) were removed (Power et al., 2014). All participants with greater than 20% of the volumes removed were excluded from further analysis. Finally, 37 mild-WMH subjects and 29 non-WMH subjects were enrolled for further functional analysis.

### Functional Connectivity Strength (FCS) and Fractional Amplitude of Low Frequency Fluctuation (fALFF)

Fractional amplitude of low frequency fluctuation (fALFF) and functional connectivity strength (FCS) was calculated using DPABI software. Briefly, for a given voxel, the time series was first converted to the frequency domain using a fast Fourier transform. The square root of the power spectrum was computed and then averaged across a predefined frequency interval. This averaged square root was termed amplitude of low frequency fluctuation (ALFF) at the given voxel (Zang et al., 2007). fALFF is the fraction of ALFF in a given frequency band to the ALFF over the entire frequency range detectable in the given signal (Zou et al., 2008). Specifically, the enrolled subjects’ fMRI images without scrubbing (Yan et al., 2013) and bandpass filtering were used to calculate fALFF. FCS was calculated for all enrolled subjects’ fMRI images following scrubbing. For each participant, Pearson correlations were computed between a given voxel and all other voxels within a GM mask. In addition, a threshold of 0.25 was set to remove the weak correlations possibly arising from signal noise (Buckner et al., 2009). The mean coefficient of a given voxel with all voxels was defined as the FCS of this voxel.

### Statistical Analysis

Statistical analysis of demographic and clinical data was performed using the Statistical Package for the Social Sciences, version 22 (SPSS 22, Chicago, IL, United States). Demographic and clinical data were compared between subjects in the mild-WMH and non-WMH groups by two-sample *t*-tests and χ^2^ tests. TBSS indices were compared by two-sample *t*-tests using FSL. Corrected *P* ≤ 0.05 was considered statistically significant.

For GM morphometric analyses (i.e., cortical thickness and surface area) and functional analysis (i.e., fALFF and FCS), we used a GLM to first determine whether there was a significant age × group interaction. If not, the main group effect was then assessed after excluding the interaction term (Engqvist, 2005). All these statistical analyses were implemented using Surfstat. The random field theory (RFT) correction was applied to correct for multiple comparisons, and clusters surviving an FWE-corrected *P* < 0.005 were considered significant. The group × age interaction on fALFF and FCS was applied across GM voxels; the interaction on cortical thickness and surface area was applied across cortical vertices. In the linear model, sex, education, age, and group were included as fixed factors. For the remaining voxels or vertices showing no significant group × age interaction, we tested the main group effect after removing the group × age interaction term while taking age, sex, and education as covariates.

### Spatial Correlation Analysis

We performed a spatial correlation analysis of the statistical maps among the indices. For GM morphometric analyses, we calculated the spatial correlation between *t* maps of cortical thickness and surface area, which was done separately for the interaction and group effects. For functional analyses, we calculated the spatial correlation between *t* maps of FCS and fALFF, which was also done separately for the interaction and group effects. Considering the huge number of voxel/vertex and the spatial autocorrelation in the *t* maps, we used BrainSMASH (Burt et al., 2020) to generate 10,000 surrogate maps that preserve spatial autocorrelation to perform permutation correction (corrected *P* < 0.05).

## Results

### Group × Age Interaction Effect on Cortical Thickness

**FIGURE 2:**
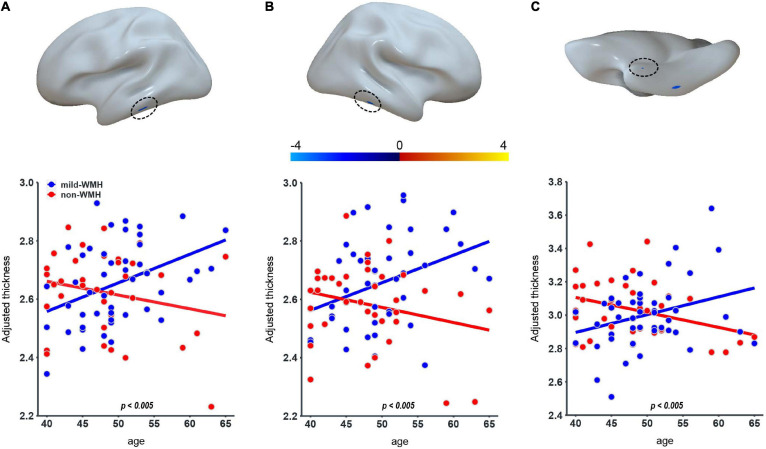
Group × age interaction effect on cortical thickness. Three clusters on the left inferior temporal gyrus **(A)**, the right inferior temporal gyrus **(B)**, and the right perirhinal entorhinal cortex **(C)** showed significant group × age interaction.

Regarding cortical thickness, there were three clusters showing a significant group × age interaction (Figure 2): the left inferior temporal gyrus (cluster 1: *t* = −2.997, *P* = 0.00365), the right inferior temporal gyrus (cluster 2: *t* = −3.008, *P* = 0.00354), and the right perirhinal entorhinal cortex (cluster 3: *t* = −2.903, *P* = 0.0048). The significant interaction indicated a group difference in the slopes between cortical thickness and age. The scatter plots revealed that as age increased, the cortical thickness of these clusters increased in the mild-WMH group but decreased in the non-WMH group. Based on the *post hoc* analysis, the cortical thickness of cluster 1 showed a significant positive correlation with age in the mild-WMH group (*r* = 0.27, *P* = 0.039) but not in the non-WMH group (*r* = −0.22, *P* = 0.26). No significant correlation was observed between cortical thickness and age in the other two significant clusters.

No significant cluster was observed for the group × age interaction on surface area.

### Group Effect on Cortical Thickness and Surface Area

**FIGURE 3:**
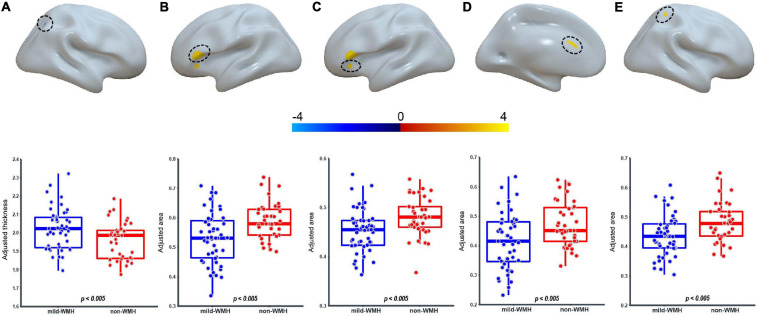
Group effect on cortical thickness and surface area. For the CT, one cluster on the right superior parietal lobule **(A)** showed significant group difference. For the surface area, four clusters on the Broca’s area **(B)**, the left pars orbitalis **(C)**, the left anterior cingulate cortex **(D)**, and the right superior parietal lobule **(E)** showed significant group difference.

Regarding cortical thickness, there was one significant cluster around the right superior parietal lobule (*t* = −2.935, *P* = 0.00436; Figure 3A), with the mild-WMH group showing greater cortical thickness than the non-WMH group.

Regarding surface area, four clusters showed significant group effects (Figures 3B–E). They were located around Broca’s area (cluster 1: *t* = 3.403, *P* = 0.00105), the left pars orbitalis (cluster 2: *t* = 3.006, *P* = 0.00355), the left anterior cingulate cortex (cluster 3: *t* = 3.028, *P* = 0.00332), and the right superior parietal lobule (cluster 4: *t* = 2.950, *P* = 0.0418). In these clusters, the mild-WMH group consistently showed a smaller surface area than the non-WMH group.

### Group × Age Interaction Effect and Group Effect on FA and MD

There was no significant age × group interaction or group effect on TBSS parameters (i.e., FA and MD) between the two groups.

### Group × Age Interaction Effect on fALFF and FCS

**FIGURE 4:**
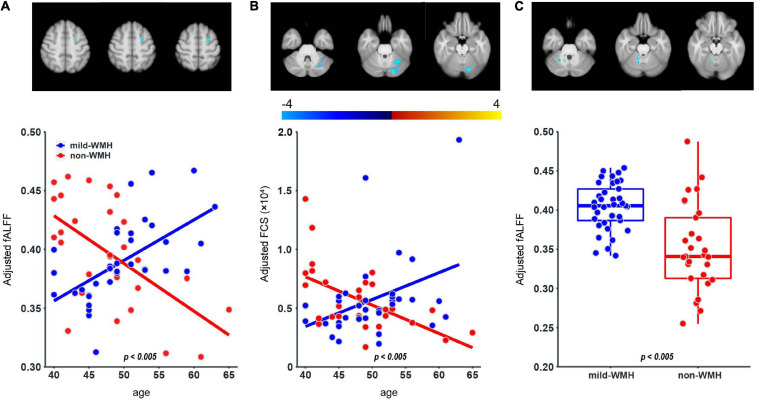
Group × age interaction effect on fALFF and FCS, and group effect on fALFF. One cluster on the left superior frontal gyrus **(A)** showed significant group × age interaction in the fALFF; one cluster on the left cerebellum crus I **(B)** showed significant group × age interaction in the FCS. For the fALFF, one cluster on the right cerebellum lobuleV **(C)** showed significant group effect.

Regarding fALFF, one cluster showed a significant group × age interaction (Figure 4A), which was located around the left superior frontal gyrus (*t* = −5.643, *P* = 4.81 × 10^–7^). The scatter plot for this cluster revealed that fALFF increased in the mild-WMH group but decreased in the non-WMH group with increasing age. Specifically, fALFF showed a significant positive correlation with age in the mild-WMH group (*r* = 0.59, *P* < 0.001) but a significant negative correlation in the non-WMH group (*r* = −0.59, *P* < 0.001).

Regarding FCS, there was one cluster on left cerebellum crus I, showing a significant group × age interaction (*t* = −4.074, *P* = 1.38 × 10^–4^; Figure 4B). Similar to fALFF above, FCS of this cluster increased in the mild-WMH group but decreased in the non-WMH group as age increased, and FCS showed a significant positive correlation with age in the mild-WMH group (*r* = 0.39, *P* = 0.017) but a negative correlation in the non-WMH group (*r* = −0.57, *P* < 0.005).

### Group Effect on fALFF

Regarding the fALFF, there was one cluster showing a significant group effect (Figure 4C), which was located around the right cerebellum lobule V (*t* = −6.114, *P* = 7.51 × 10^–8^). Specifically, the mild-WMH group had a larger fALFF than the non-WMH group.

All results of group × age interaction and main group effects are summarized in Table 2.

**TABLE 2:** The results of group effect and group × age interaction.

		**Group effect**			**Group × age interaction effect**		
		**Position**	***T*-Value**	***P*-value**	**Position**	***T*-Value**	***P*-value**
3D T1	Cortical thickness	Right superior parietal lobule	–2.935	0.00436	Left inferior temporal gyrus	2.997	0.00365
					Right inferior temporal gyrus	–3.008	0.00354
					Right Perirhinal entorhinal cortex	–2.903	0.0048
	Surfer area	Broca’s area	3.403	0.00105	NS*		
		Left pars orbitalis	3.006	0.00355			
		Left anterior cingulate cortex	3.028	0.00332			
		Right superior parietal lobule	2.950	0.0418			
rs-fMRI	fALFF	Right cerebellum lobule V	–6.114	7.51 × 10^–8^	Left superior frontal gyrus	–5.643	4.81 × 10^–7^
	FCS	NS			Left cerebellum crus I	4.074	1.38 × 10^–4^
DTI	FA	NS			NS		
	MD	NS			NS		

*NS, not significant.

### Spatial Correlation Analysis of Statistical Maps Among Indices

There are no significant correlation (corrected *P* < 0.05) between indices in either group effect or group × age interaction (Table 3).

**TABLE 3:** The results of spatial correlation of statistical maps between indices.

		**Group effect**		**Group × age interaction effect**	
		***R*-Value (Pearson)**	*P*_perm_-value***	***R*-Value (Pearson)**	***P*_perm_-value**
3D T1*	Left-hemisphere cortex	0.0081	0.948	0.063	0.619
	Right-hemisphere cortex	–0.18	0.226	–0.22	0.149
rs-fMRI**		0.028	0.253	0.14	0.230

*Spatial correlation between *t* maps of cortical thickness and surface area; the correlation was performed for each hemisphere separately due to the requirement of permutation correction algorithm. **Spatial correlation between *t* maps of FCS and fALFF. ***P-value after permutation correction.

## Discussion

As a common accompaniment of aging, WMH can be symptomatic or completely asymptomatic. It has been suggested that if the severity or amount of WMH is above a certain threshold, the WMH is likely associated with cognitive impairment and psychomotor abnormalities (Sachdev et al., 2005). It is therefore valuable to ascertain the neuroimaging profile outside of the WMH territory in asymptomatic adults with very early-stage WMH, as this would provide insight into the pathogenesis of WMH.

Cortical thickness is typically decreased during healthy aging (Hutton et al., 2009). In our study, we observed a significant group × age interaction in the left inferior temporal gyrus, the right inferior temporal gyrus and the right perirhinal entorhinal cortex between the asymptomatic mild-WMH group and the control group, suggesting a group difference in the age-related changes in cortical thickness. Additionally, we found greater cortical thickness in the right superior parietal lobule in the mild-WMH group than in the control group. It has been reported that MCI subjects with WMH exhibited cortical thinning in the frontal, perisylvian, basal temporal, and posterior cingulate regions compared with normal subjects (Kim et al., 2014). Another study found that MCI subjects with WMH exhibited cortical thinning in the inferior frontal and orbitofrontal gyri, anterior cingulate gyrus, insula, superior temporal gyrus, and lingual gyrus (Seo et al., 2010). It is possible that the age-related difference and higher cortical thickness observed in the asymptomatic mild-WMH group reflect compensatory effects for maintaining normal cognitive function.

In contrast, the surface area showed a decreased pattern in the asymptomatic mild-WMH group. This may reflect accelerated neural degeneration in WMH patients even in this very early stage. It is possible that the degree of regional atrophy does not reach the particular threshold that causes any clinical symptoms. Further studies with a larger sample size and more elaborate neurological scales are needed to evaluate this speculation.

Regarding the functional measures, the left superior frontal gyrus showed a significant group × age interaction on fALFF, and the left cerebellum crus I showed a significant group × age interaction on FCS. Both of them were increased in the mild-WMH group but decreased in the non-WMH group with increasing age. In addition, the asymptomatic mild-WMH group showed higher fALFF in the right cerebellum lobule V. Compatible with our current findings, reduced functional connectivity (FC) between the cerebellum and several supratentorial regions was found in young neurologically asymptomatic WMH adults (Keřkovský et al., 2019). Another study found that the WMH group showed a significant decrease in ALFF in the left parahippocampal gyrus (PHG) and increased ALFF in the left inferior semi-lunar lobule and right superior orbital frontal gyrus (SOFG) but increased FC between the right insular region and the right SOFG and between the right calcarine cortex and the left PHG (Cheng et al., 2017). Notably, the functional measures differed among these studies, making it difficult for a direct comparison. It is likely that the observed anomalies, either age-related or not, represent compensatory effects to achieve normal levels of cognition and motor control in asymptomatic mild-WMH subjects.

Finally, no significant anomalies in FA and MD were observed in the asymptomatic WMH patients. This may be related to the very small effect size of WM changes and limited statistical power of our study due to the sample size.

In our study, the observed age effect above may simply reflect a WMH effect, given that the older people likely have more and larger WMH. To evaluate this possibility, we tested the correlation between the WMH size with age across out patients but found no significant correlation, therefore ruling out such possibility.

We also explored the relationship of the statistical maps among the imaging indices but found no significant correlations, suggesting the independence of these imaging indices.

Several limitations should be addressed. First, our sample size was relatively small, which could limit the statistical power for identifying changes with small effect sizes (which possibly accounted for the negative results with FA/MD). Next, the neurological scales for determining the lack of symptoms were simply based on the MMSE and ADL, and more comprehensive neurological scales could be applied in future studies. Finally, the imaging protocol could be improved in future studies. For example, the acquisition time of resting-state functional images in our study was relatively short, and longer scanning has been encouraged in the field.

## Conclusion

In conclusion, our study demonstrated both structural and functional anomalies of a set of specific GM regions in community-dwelling asymptomatic adults with very early-stage WMH. These findings provide novel data regarding very early-stage WMH and enhance understanding of the pathogenesis of WMH.

## Data Availability Statement

The raw data supporting the conclusions of this article will be made available by the authors, without undue reservation.

## Ethics Statement

The studies involving human participants were reviewed and approved by the Research Ethics Committee of Qilu Hospital (Qingdao), Cheeloo College of Medicine, Shandong University. The patients/participants provided their written informed consent to participate in this study.

## Author Contributions

XXM and XSM conceived and designed the research. SG performed the experiments. SG, SD, QR, ZH, and YL collected the data. XK and WW performed the imaging analysis. SG and XK wrote the manuscript. GG reviewed and edited the manuscript. All authors contributed to the article and approved the submitted version.

## Conflict of Interest

The authors declare that the research was conducted in the absence of any commercial or financial relationships that could be construed as a potential conflict of interest.

## Publisher’s Note

All claims expressed in this article are solely those of the authors and do not necessarily represent those of their affiliated organizations, or those of the publisher, the editors and the reviewers. Any product that may be evaluated in this article, or claim that may be made by its manufacturer, is not guaranteed or endorsed by the publisher.

## Footnotes

1
http://www.nitrc.org/projects/panda

2
https://fsl.fmrib.ox.ac.uk/fsl/fslwiki/FSL

3
http://rfmri.org/dpabi

4
http://www.fil.ion.ucl.ac.uk/spm
